# The Usefulness of Trivalent Phosphorus for the Synthesis of Dendrimers

**DOI:** 10.3390/molecules26020269

**Published:** 2021-01-07

**Authors:** Anne-Marie Caminade, Kathleen I. Moineau-Chane Ching, Béatrice Delavaux-Nicot

**Affiliations:** 1 Laboratoire de Chimie de Coordination du CNRS, UPR 8241, 205 Route de Narbonne, BP 44099, CEDEX 4, 31077 Toulouse, France; kathleen.chane@lcc-toulouse.fr (K.I.M.-C.C.); beatrice.delavaux-nicot@lcc-toulouse.fr (B.D.-N.); 2 LCC-CNRS, Université de Toulouse, CNRS, Toulouse, France

**Keywords:** dendrimers, phosphines, Staudinger reaction, phosphoramidites, alkylation

## Abstract

Dendrimers are hyperbranched macromolecules, which are synthesized step-by-step by the repetition of a series of reactions. While many different types of dendrimers are known, this review focusses on the use of trivalent phosphorus derivatives (essentially phosphines and phosphoramidites) for the synthesis of dendrimers. The first part presents dendrimers constituted of phosphines at each branching point. The other parts display the use of trivalent phosphorus derivatives during the synthesis of dendrimers. Different types of reactions have been applied to phosphines. The very first examples of phosphorus-containing dendrimers were obtained by the alkylation of phosphines. Then, several families of dendrimers were elaborated by reaction of phosphoramidites. Such a type of reaction is the base of the solid phase synthesis of oligonucleotides; it has been applied in particular for the synthesis of dendrimers constituted of oligonucleotides. Finally, the Staudinger reaction between phosphines and azides afforded different families of dendrimers, and was at the origin of accelerated methods of synthesis of dendrimers. Besides, the reactivity of the P=N-P=S linkages created by this reaction led to very original dendritic structures.

## 1. Introduction

**Figure 1:**
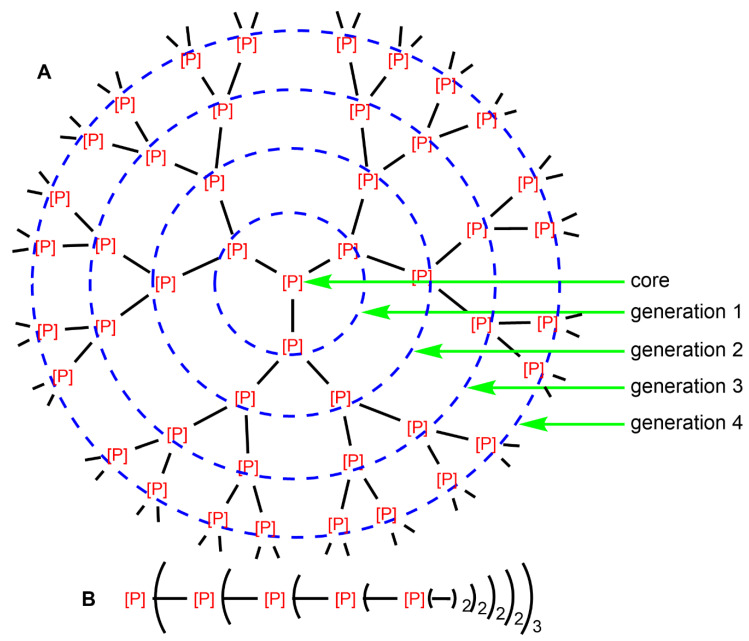
Two types of schematized structure of dendrimers (generation 4) having phosphorus atoms at each branching point, either of type phosphine, or issued from the reaction of phosphines. (**A**): Full structure; (**B**): Linear representation of the same dendrimer.

Dendrimers are macromolecules constituted of regular branching layers, obtained by the step-by-step association of identical monomers. Each time the number of terminal functions is multiplied, generally by 2, a new “generation” is created. Most dendrimers are purely organic, with nitrogen as a branching element. However, several dendrimers constituted of main group elements at the branching points, essentially phosphorus and silicon, are also well-known and have been reviewed very early [1], and more recently [2]. Many different types of phosphorus-containing dendrimers have already been synthesized, since the pioneering works carried out in the early nineties [3]. One of the main advantages of phosphorus-containing dendrimers (Figure 1) over other types of dendrimers is the possibility to use ^31^P NMR for both monitoring the course of the synthesis and assessing the completeness of the reactions. The purity of the isolated dendrimers can be also ascertained [4]. The most studied type of phosphorus dendrimers is based on the phosphorhydrazone linkage O-C_6_H_4_-CH=N-N(Me)P(S) [5]. These phosphorhydrazone dendrimers have been used in many different fields, depending on the type of functions they bear on the surface, in particular for catalysis, for the elaboration of nanomaterials, and for biology. However, no phosphine is involved for their synthesis [6], thus they will not be displayed in this review.

Other types of phosphorus dendrimers have been proposed, in particular those synthesized using phosphines (the official IUPAC nomenclature for trivalent phosphorus atoms is phosphane, but phosphine is more widely used). Phosphines are well-known ligands in coordination chemistry, in particular for catalysis, but their use for the synthesis of dendrimers is by far less documented. A few dendrimers are entirely constituted of phosphines at the branching points, but several other phosphorus-containing dendrimers are synthesized by methods involving phosphines at each step. The most important methods of synthesis for this second type of dendrimers elaborated with the help of tricoordinated phosphorus derivatives are the alkylation of phosphines, the use of phosphoramidites followed by oxidation, and the Staudinger reaction of phosphines with azides [7]. These different methods of synthesis will be described below, starting with dendrimers entirely constituted of phosphines or phosphites at the branching points, followed by the alkylation of phosphines, then the use of phosphoramidites. In the final part, the Staudinger reaction for the creation of P=N-P=S linkages will be described, as well as part of the reactivity carried out on these linkages, in particular for the elaboration of highly sophisticated dendritic structures.

## 2. Dendrimers Constituted of a Trivalent Phosphorus Derivative at Each Branching Point

**Scheme 1:**
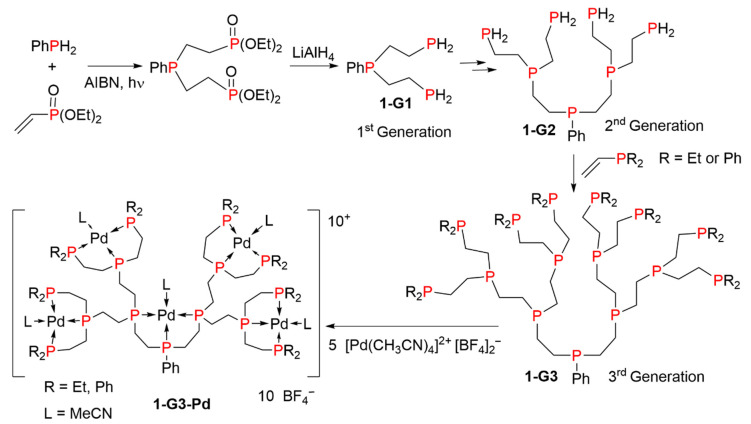
The first example of dendrimers constituted of phosphines at each branching point.

The first example of dendrimers constituted of phosphines at each branching point was a relatively small compound, as the branches were constituted of only three bonds, but it was a third-generation dendrimer. This family of compounds was synthesized by the repetition of the sequential addition of diethyl vinylphosphonate to primary phosphines, in the presence of AIBN (azobisisobutyronitrile), followed by reduction with lithium aluminum hydride. The reaction of the third generation dendrimer **1-G3** with [Pd(CH_3_CN)_4_][BF_4_]_2_ afforded the complex **1-G3-Pd** shown in Scheme 1. This complex was used as a catalyst in the electrochemical reduction of CO_2_ to CO. This is one of the very first examples of the use of dendrimers in catalysis [8].

**Scheme 2:**
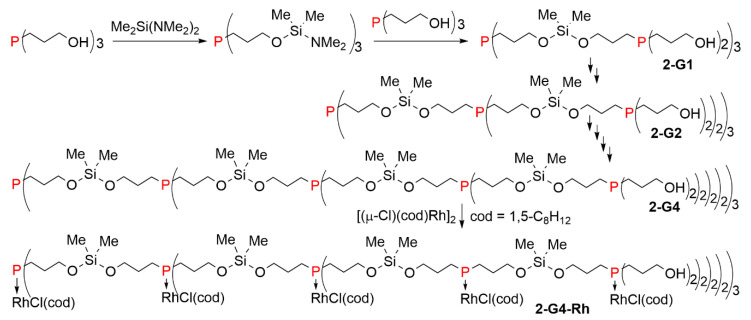
Synthesis of a large phosphine dendrimer, and its complexation with rhodium.

**Figure 2:**
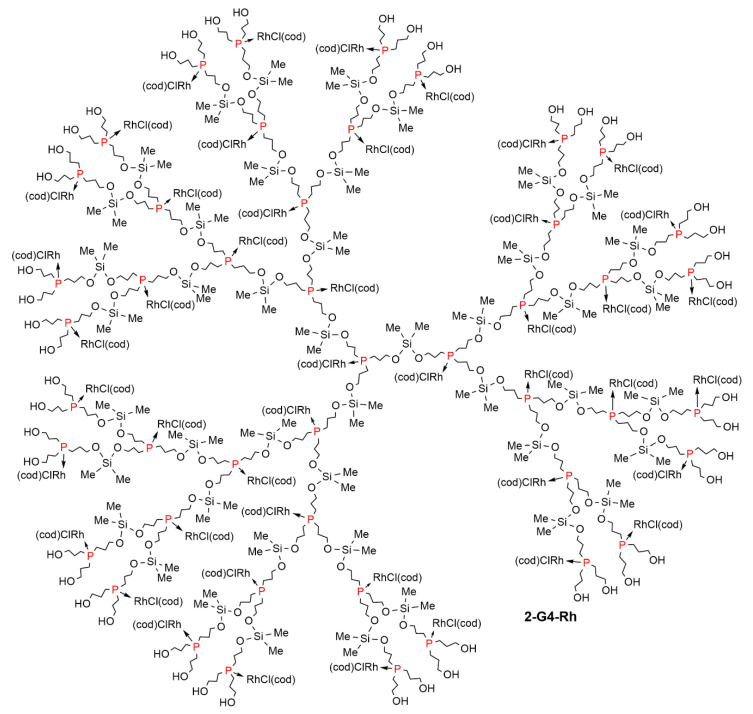
Full chemical structure of the dendritic complex **2-G4-Rh**, synthesized as shown in Scheme 2.

A larger dendrimer was obtained by the controlled addition of tri(hydroxypropyl)phosphine to 3 equivalents of bis(dimethylamino)dimethylsilane. The resulting compound was added to 3 equivalents of tri(hydroxypropyl)phosphine, resulting in the synthesis of the first-generation dendrimer **2-G1**. By repeating the sequence of reactions with Me_2_Si(NMe_2_)_2_ and P[(CH_2_)_3_OH]_3_, the second, third, and fourth generations of the dendrimer were prepared (**2-G1** to **2-G4**). All generations, including the fourth one, reacted with μ-chloro-(1,5-cyclooctadiene)rhodium (I) dimer, [(μ-Cl)(cod)Rh]_2_ to afford the Rh-complex of the dendrimer **2-G4-Rh** shown in Scheme 2 for its linear form and in Figure 2 for the full structure. The same complexes were also obtained by using the complex Rh(cod)(Cl)P[(CH_2_)_3_-OH]_3_ instead of P[(CH_2_)_3_OH]_3_ during the synthesis of the dendrimers. These dendrimer complexes were found efficient catalysts for olefin hydrogenation under standard conditions, using decene as the substrate. The turnover number and frequency were found similar to that of the monomeric complex [9].

**Scheme 3:**
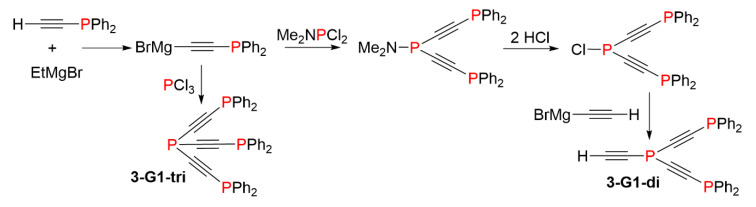
Small acetylenic phosphine dendrimers.

Very small phosphine dendrimers (first generations) having acetylene as branches were synthesized. The reaction of bromo[(diphenylphosphino)ethynyl] magnesium with PCl_3_ afforded the compound **3-G1-tri**. This compound was characterized by the presence of two signals in ^31^P NMR, a quadruplet at −87.5, and a doublet at −31.4 ppm (J_PP_ = 4.8 Hz), corresponding to the phosphine at the core, and to PPh_2_ groups, respectively. Another attempt was made by reacting the same Grignard reagent with Me_2_NPCl_2_, followed by the reaction with HCl, then with BrMgC≡CH, to afford compound **3-G1-di** (Scheme 3). This compound is potentially suitable to continue the growing of the dendrimer, but it was isolated in too small a quantity to perform the next step [10].

**Scheme 4:**
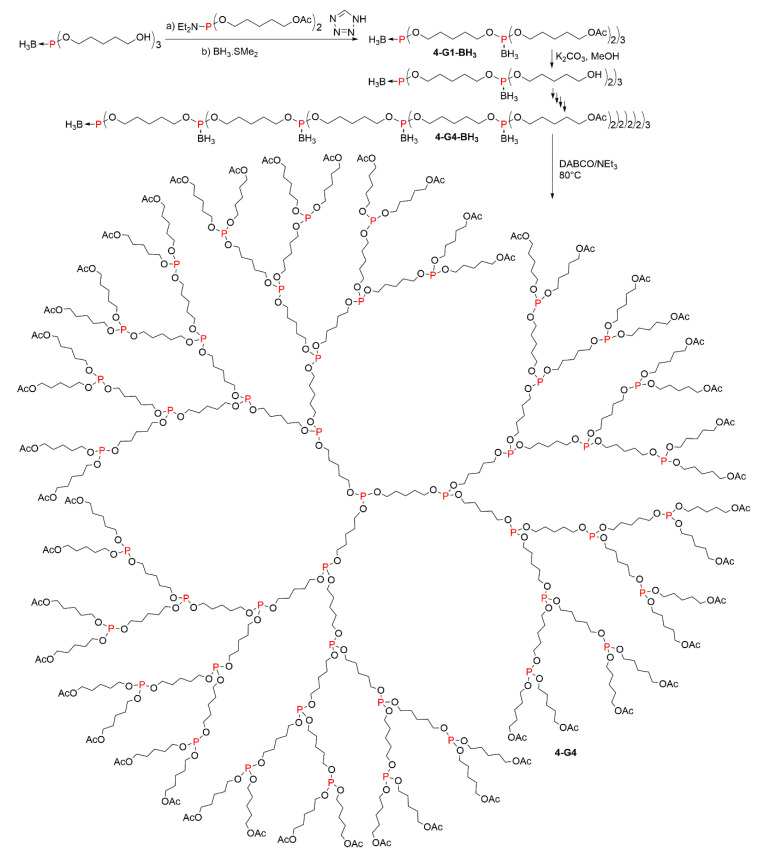
Synthesis of boranophosphate triesters and phosphite-based dendrimers.

A series of phosphite dendrimers was synthesized by deprotection in the final step of boranophosphate triesters. The synthesis begun from tris(5-hydroxypentyl)boranophosphate, used as core. The synthetic strategy consisted in the first step in a phosphitylation of alcohol groups with the phosphoramidite Et_2_N-P(O(CH_2_)_5_OAc)_2_, followed by the addition of BH_3_.SMe_2_. Such a methodology will be described in more detail in Section 4 of this review. The second step was the cleavage of the terminal acetate functions with saturated K_2_CO_3_ in methanol, to generate alcohol terminal functions on which the two-step synthesis was carried out again. The synthesis was carried out up the fourth generation **4-G4-BH_3_** (Scheme 4). All compounds displayed in ^31^P NMR a very broad quartet at 115 ppm (^1^J_31P–11B_ ≈ 124 Hz). Removal of BH_3_ was carried with DABCO (1,4-diazabicyclo(2.2.2)octane) in NEt_3_ at 80 °C, to afford the phosphite dendrimers up to the fourth generation **4-G4**. The ^31^P NMR spectra displayed only a single signal at about 139 ppm for all the phosphite dendrimers, even **4-G4** [11].

**Scheme 5:**
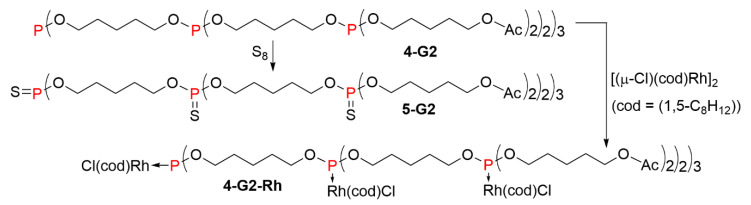
Oxidation with sulfur and complexation with rhodium of a 2nd generation phosphite dendrimer.

The second generation of the phosphite dendrimer **4-G2** was reacted with an excess of elemental sulfur to afford the corresponding thiophosphate **5-G2** (Scheme 5). The ^31^P NMR spectrum displayed three distinct signals at 68.50, 68.56, and 68.60 ppm in the 6:1:3 ratio, respectively. Complexation experiments were carried out on **4-G2** with the rhodium(I) dimer [(μ-Cl)(cod)Rh]_2_, affording the dendritic complex **4-G2-Rh**. The complexation was characterized in the ^31^P NMR spectrum by the presence of a doublet at 117.9 ppm (^1^J_P-Rh_ = 240.8 Hz). Complexation of dendrimer **4-G2** was also carried out with 5 equivalents of [PdCl_2_(cod)]. Complexation of one Pd necessitates two phosphite groups, which can occur in different ways inside the dendrimer structure, thus the formation of a single defined product appears unlikely, and is not represented here. However, ^31^P NMR spectroscopy confirmed the complexation of all phosphite groups, with the appearance of a single signal at 94.6 ppm, to be compared to 139.6 ppm for the non-complexed phosphite dendrimer **4-G2** [11].

## 3. Dendrimers Synthesized by Alkylation of Phosphines

**Scheme 6:**
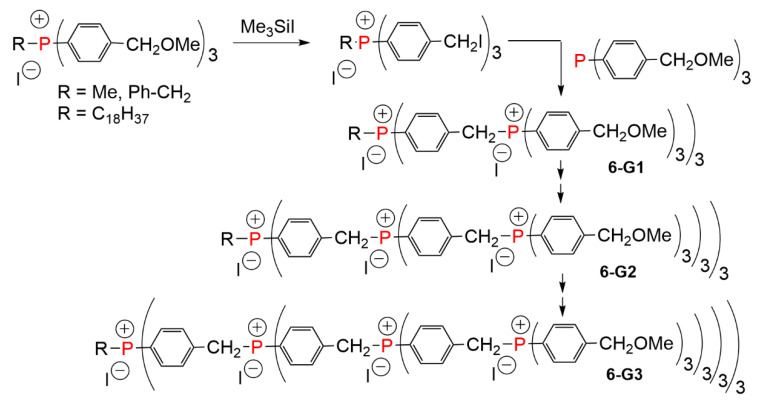
Alkylation of phosphines for the synthesis of phosphonium dendrimers up to the third generation.

The very first example of phosphorus-containing dendrimers was based on quaternary phosphonium salts at each branching point. The core, as well as the branching units, were derived from *tris*(*p*-methoxymethylphenyl) phosphine. Successive generations were obtained via treatment of the benzylic alkyl ether with iodotrimethylsilane, affording benzylic iodide. The second step was the quaternization of *tris*(*p*-methoxymethylphenyl) phosphine with the benzylic iodide, to afford the subsequent generation (Scheme 6). In the first examples, the R group at the core of compound **6-G2** was either methyl or benzyl [3]. In the following examples, an octadecyl chain was an additional R group, and the dendrimers were synthesized up to the third generation, having 81 terminal functions [12].

**Scheme 7:**
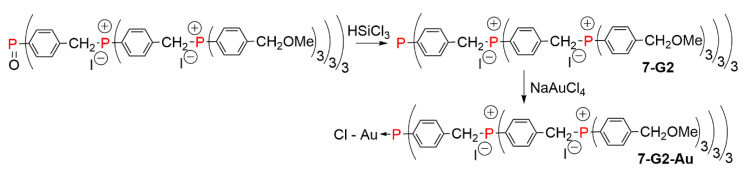
Phosphonium dendrimers having a phosphine oxide, a phosphine, or a phosphine complexing gold at the core.

**Figure 3:**
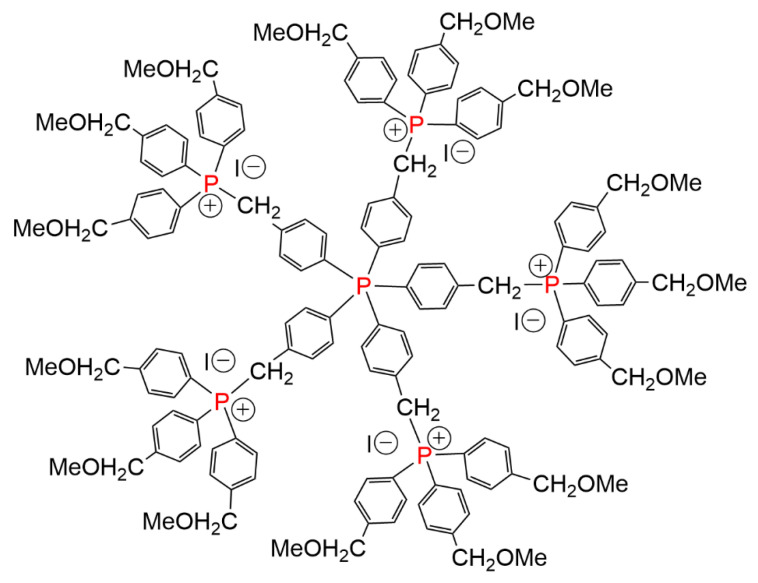
Phosphonium dendrimer built from a quinque-directional phosphorane core.

The same family of dendrimers was synthesized starting from a phosphine oxide at the core, up to the second generation. The phosphine oxide was then reduced with HSiCl_3_, to afford the free phosphine **7-G2**. In a last step, the phosphine was used for the complexation of gold, affording **7-G2-Au** (Scheme 7) [13]. In another experiment, a phosphorane was used as a very original quinque-directional core for the synthesis of the first generation phosphonium dendrimer (Figure 3) [13].

## 4. Phosphoramidite Reagents for the Synthesis of Dendrimers

**Scheme 8:**
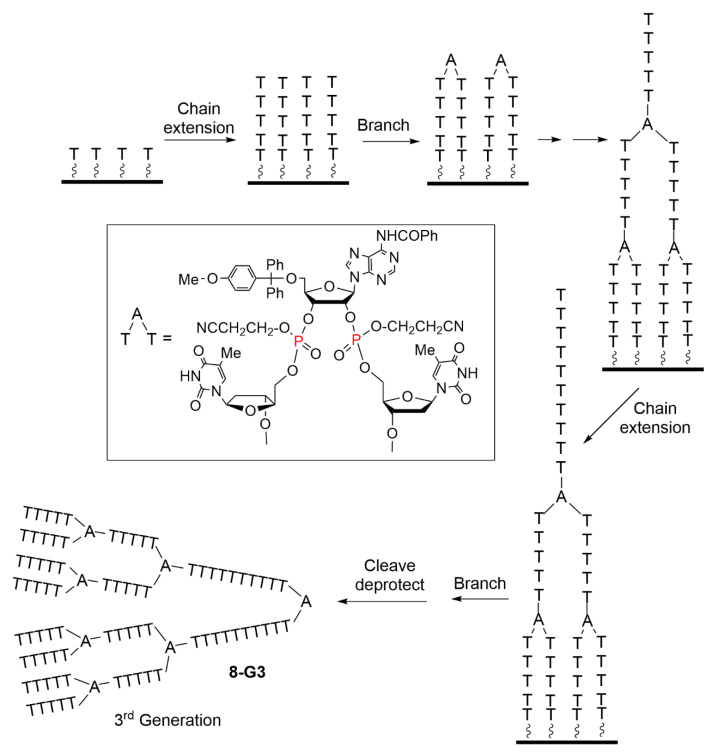
Synthesis via a convergent process of nucleic acid dendrimers based on thymidine (T) for the branches and adenosine (A) 2′,3′-bis(phosphoramidite) reagent for the branching points.

Phosphoramidite reagents are at the origin of the dramatic development of automated synthesis of DNA fragments from a solid matrix, which has revolutionized both biology and medicine [14]. However, this method, which is perfectly suitable for the growing of the linear chain of DNA fragments, necessitates some adaptations at the level of the phosphoramidites to become suitable for the synthesis of dendrimers. The first examples in this field concerned the synthesis of nucleic acid dendrimers. The first step was the classical construction of short oligonucleotides with an automated DNA synthesizer on a solid surface functionalized with long chain alkylamine, and using thymidine (T). The second step consisted in coupling two adjacent nucleotide chains with a bifunctional adenosine (A) 2′,3′-bis(phosphoramidite), affording after coupling and oxidation the adenosine diphosphate shown in the insert of Scheme 8. A low concentration of bis(phosphoramidite) was employed. In these conditions, only a fraction of the 5′-hydroxyl groups was phosphitylated, hence favoring the branching. However, some 5′-hydroxyl groups remained unreacted. Repetitive chain elongation and branching steps generated successive generations in a convergent growth, from the surface to the core. The last step was the cleavage from the solid support to generate the nucleic acid dendrimers **8-G3** (Scheme 8) [15].

**Scheme 9:**
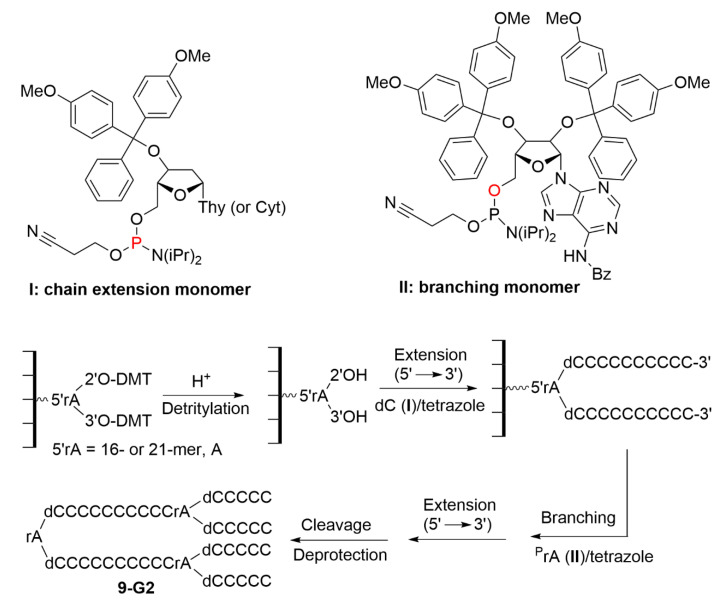
Structure of phosphoramidite reagents used for chain extension (**I**) and branching (**II**), and the divergent synthesis of nucleic acid dendrimers **9-G2**, in the solid phase.

Another report concerned an analogous method, but was carried out using a divergent process. The divergent-growth strategy should remove the steric and geometric constraints encountered in the convergent approach, especially when coupling the neighboring oligomers as shown in Scheme 8. In this new experiment, the chains were constituted of cytosine (C), whereas the branching units comprised adenosine (A). One phosphoramidite was used for chain extension (compound **I** in Scheme 9), and another one was used as a branching unit (compound **II** in Scheme 9). The synthesis starts with the adenine branching monomer (**II**), eventually linked to the solid support via a 16- or 21-mer. The average coupling yield of the bulky branching ribonucleoside (compound **II**) was low, ca. 65–95%, when compared to the >95% yield obtained with the linear deoxynucleoside monomer (compound **I**). The main reason is that the 5′-amidite moiety in compound **II** is less accessible to coupling, due to the steric hindrance in its environment. After cleavage from the solid support and deprotection, the second generation dendrimer **9-G2** was finally obtained, as shown in Scheme 9 [16].

**Figure 4:**
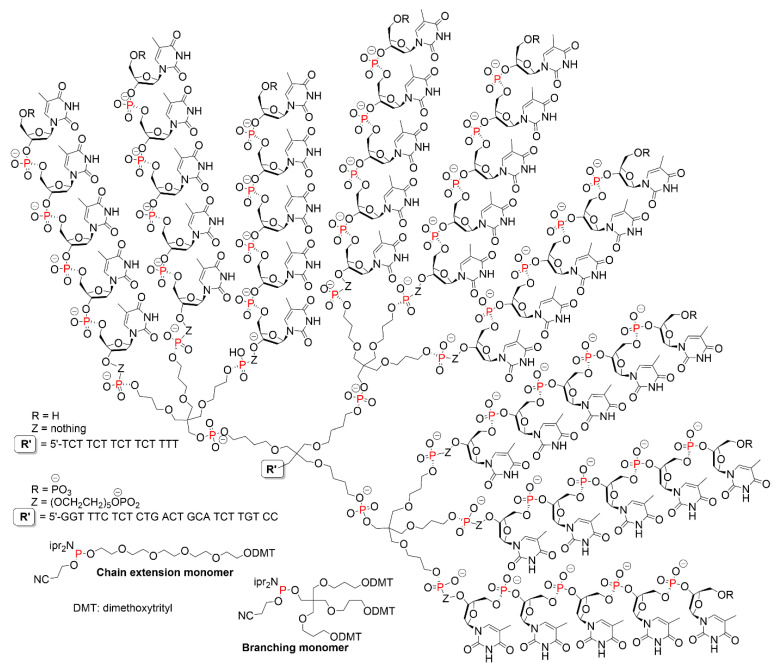
Phosphoramidite functionalized with pentaerythritol structure for branching and phosphoramidite functionalized with an oligoethyleneoxide chain to reduce the density of packing. Structure of dendrons bearing an oligonucleotide at the core and pentathymidine on the surface.

Other types of oligonucleotide dendrimers were synthesized by the same methods, using other phosphoramidite reagents. An example concerned a phosphoramidite functionalized with a pentaerythritol structure, to treble the number of branches at each coupling, and a phosphoramidite functionalized with an oligoethyleneoxide chain to reduce the density of packing. Several dendrimers were synthesized, bearing an oligonucleotide at the core to be used as probe, and nine pentathymidine on the surface (Figure 4). Due to the presence of this long chain oligonucleotide at the core, this compound should be described as a dendron (dendritic wedge) more than a dendrimer. These compounds were used as PCR (polymerase chain reaction) primers. A profound effect on the mobility of the PCR products was observed, as the dendrons decreased the mobility on agarose or polyacrylamide gels [17]. The same concept was then extended to larger compounds bearing up to 27 branches instead of 9, and comprising several types of oligonucleotides as branches. These compounds were associated as complementary pairs both in solution and in the solid state. Duplex stabilities with these dendritic structures were found greater than those observed with unbranched molecules of equal length [18]. Other examples of dendritic structures based on a pentaerythritol phosphoroamidite synthon and having nine antisense oligonucleotides (ODNs) as terminal functions were obtained via automated DNA synthesis. The cellular uptake of ODN-dendrimer conjugates was up to 4-fold greater than naked ODN in cancer cells, and were found effective in inhibiting cancer cell growth [19].

**Scheme 10:**
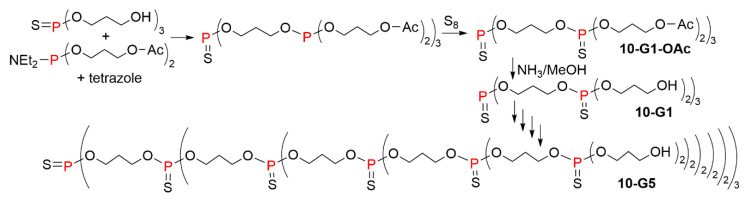
Synthesis of thiophosphate dendrimers, using a phosphoramidite as phosphitylating agent.

The use of phosphoramidite reagents was also applied to the synthesis of non-nucleotide dendrimers, in connection with the method shown in Scheme 4 for the synthesis of dendrimers having phosphines at each branching point. This method was first applied to the synthesis of thiophosphate dendrimers. The core was the tri(3-hydroxy)propyl thionophosphate. The first step of the synthesis of the dendrimers was the phosphitylation of this triol using the phosphoramidite Et_2_N-P(O-(CH_2_)_3_OAc)_2_, to generate phosphite intermediates. Sulfur addition to the phosphite dendrimers afforded the first generation dendrimer **10-G1-OAc**. The second step was the cleavage of the peripheral acetate groups, using a large excess of 30% NH_3_/MeOH, to generate alcohol functions, on which the phosphitylation with the phosphoramidite was carried out to continue the growing of the dendrimer. The synthesis was carried out up to the fifth generation **10-G5** (Scheme 10) [20].

**Scheme 11:**
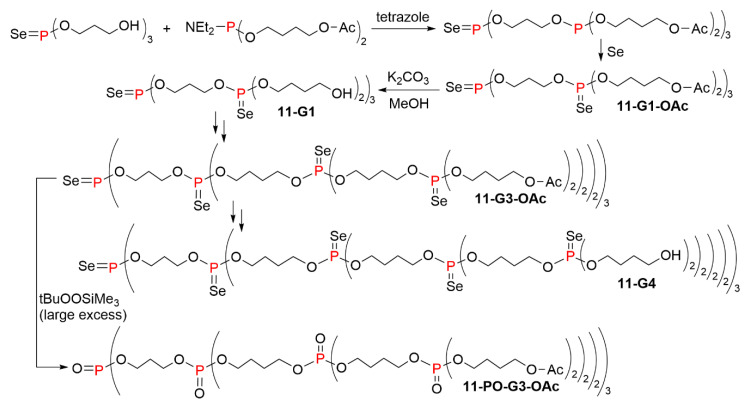
Synthesis of selenophosphate dendrimers and oxygenation to phosphate dendrimers.

The same methodology was applied to the synthesis of selenophosphate dendrimers, using black selenium instead of sulfur at the oxidation step, and a phosphoramidite with slightly longer branches, Et_2_N-P(O-(CH_2_)_4_OAc)_2_. The synthesis was carried out up to generation 4 in this case (dendrimer **11-G4**, Scheme 11). ^31^P NMR proved to be especially useful for the characterization of these dendrimers. The ^31^P{^1^H} NMR spectrum of the fourth generation **11-G4-OAc** displayed five distinct signals with the expected integration for each layer at 73.99 (1P, core), 73.72 (3P), 73.57 (6P), 73.54 (12P), 73.41 (24P) ppm. Satellite side bands with the splitting of 931 Hz for the ^31^P–^77^Se coupling were observed on each signal [21]. The complete oxygenation of this family of selenophosphate dendrimers was successfully attempted on the third generation **11-G3-OAc**, using a large excess of the peroxide t-BuOOSiMe_3_. The total conversion to the phosphate dendrimer **11-PO-G3-OAc** was observed after 3 days, with the concomitant precipitation of red selenium (Scheme 11). The reaction was monitored by ^31^P NMR, which displayed the progressive disappearance of the signals corresponding to P=Se at about 73 ppm, together with the appearance of new signals at about 0 ppm corresponding to P=O. It appeared that the reactions occurred first on the most external P=Se groups of the dendrimer. No damage to the dendrimer backbone was observed during this reaction [21].

**Figure 5:**
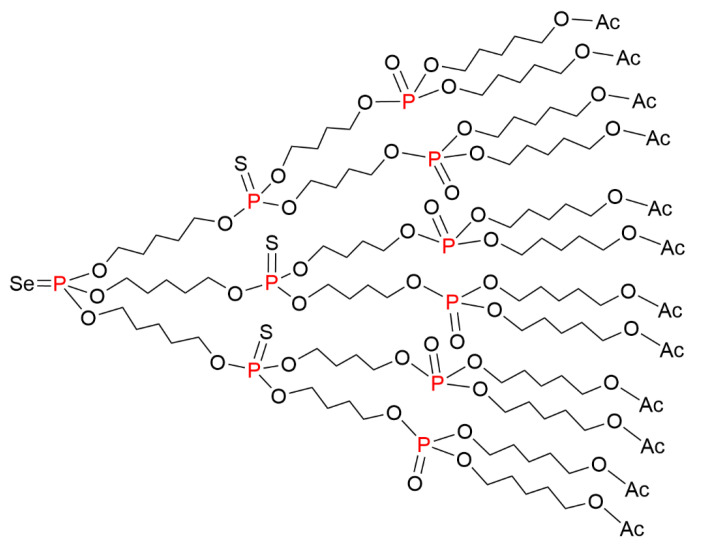
Layered dendrimers bearing three different types of branching units (P=Se, P=S, P=O).

The synthesis of dendrimers bearing three different types of branching units (P=Se, P=S, P=O) was also carried out using the same types of reactions. The starting point was a selenophosphate core. Sulfur was added to the first generation to oxidize the phosphite intermediate. The P=O groups at the level of the second generation were obtained by reaction of t-BuOOSiMe_3_ on the phosphite intermediate, to afford the dendrimer shown in Figure 5. The ^31^P NMR spectrum of this layered dendrimer displayed three very distinct signals at δ = 72.58 (P=Se), 68.72 (P=S), and -0.31 (P=O) ppm [22].

Some properties of the thiophosphate dendrimers family were reported. Generations 1 and 2 were functionalized with acyclovir on the surface, an effective drug in the treatment of HSV (Herpes Simplex Virus) infections. However, the biological properties of these dendrimers were not studied [23]. Generations 1 to 3 were functionalized with 1,3,5-benzenetricarboxylic acid derivatives [24], or with phosphonates to induce solubility in water in both cases [25]. The biological properties of the fifth generation **8-G5** were evaluated. It was shown that this compound is neither hemotoxic nor cytotoxic within the concentration range from 100 pM to 10 μM, and that it has a good biocompatibility [26].

**Figure 6:**
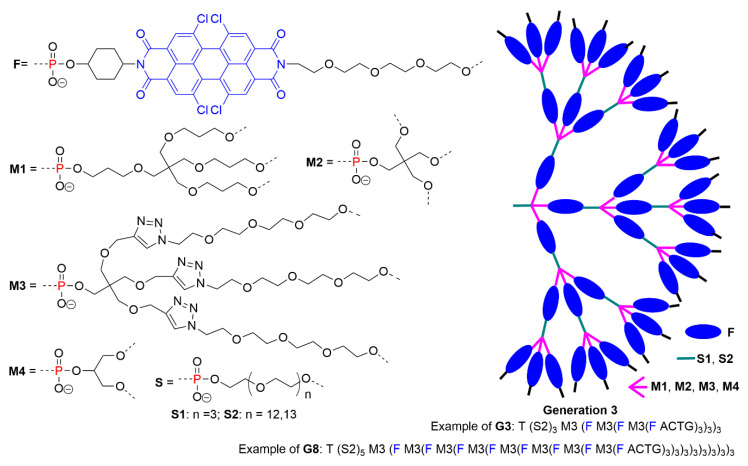
The various building blocks based on phosphoramidite reagents used for the synthesis of dendrimers built with perylenes in the branches, and schematization of the type of dendrimers obtained.

Another family of dendrimers synthesized thanks to the reactivity of especially engineered phosphoramidite reagents bearing fluorophores was elaborated. Exceptionally bright fluorescent and water-soluble dendrimers were synthesized up to the eighth generation, using the different types of fragments shown in Figure 6. Generation 8 comprised ca. 9840 fluorophores of type twisted perylenes in its structure. This compound was the brightest of the series (114 times more fluorescent than tetrachloro perylene diimide). However, the normalized brightness per dye (fluorescence intensity divided by the number of dyes, assuming value 1 for tetrachloro perylene diimide) was very low (0.012), indicating the occurrence of self-quenching phenomenon [27].

## 5. The Staudinger Reaction

### 5.1. The Staudinger Reaction for the Synthesis of Regular Dendrimers

The Staudinger reaction between a phosphine and an azide is a very clean reaction that generates only N_2_ as a by-product. However, the P=N function created by this reaction is very sensitive to water, and is easily cleaved to generate a primary amine on one side and a phosphine oxide on the other side [7]. Using this reaction for the synthesis of dendrimers necessitates first to find a way for stabilizing the P=N function. Conjugation of this function could stabilize it, thus it appeared tempting to react the phosphine not with an alkylazide, but with a thiophosphine azide, in order to create P=N-P=S linkages. Indeed, DFT calculations on this linkage demonstrated that there is a noticeable delocalization of the charge in the P=N-P=S linkage, suitable for stabilizing the P=N bond [28]. Even if it has been shown that an azide group linked to phosphorus has a different reactivity when compared to organic azides [29], the Staudinger reaction works well with such azides, as shown for the synthesis of small compounds [30]. Thus, this reaction was applied to the synthesis of dendrimers, at each generation, or at one specific layer inside the structure of dendrimers. Only the case of the synthesis of regular dendrimer incorporating this linkage at each generation will be considered here.

**Scheme 12:**
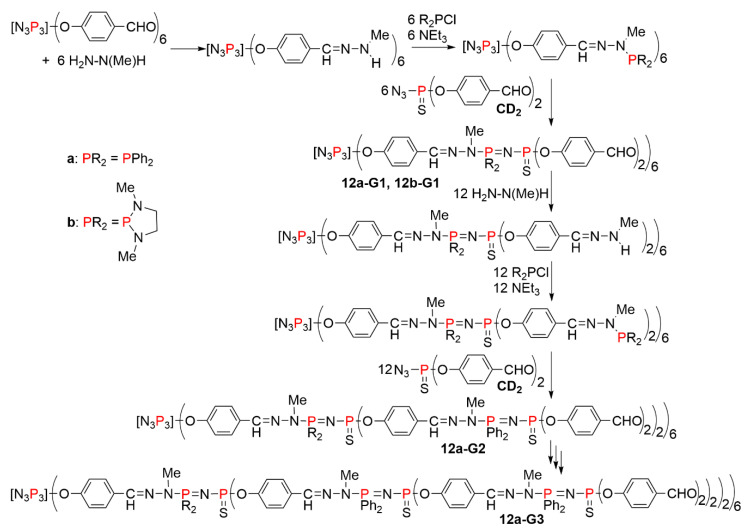
The first example of dendrimers synthesized using the Staudinger reaction between phosphines and azides at one of the three steps needed to build one generation.

**Figure 7:**
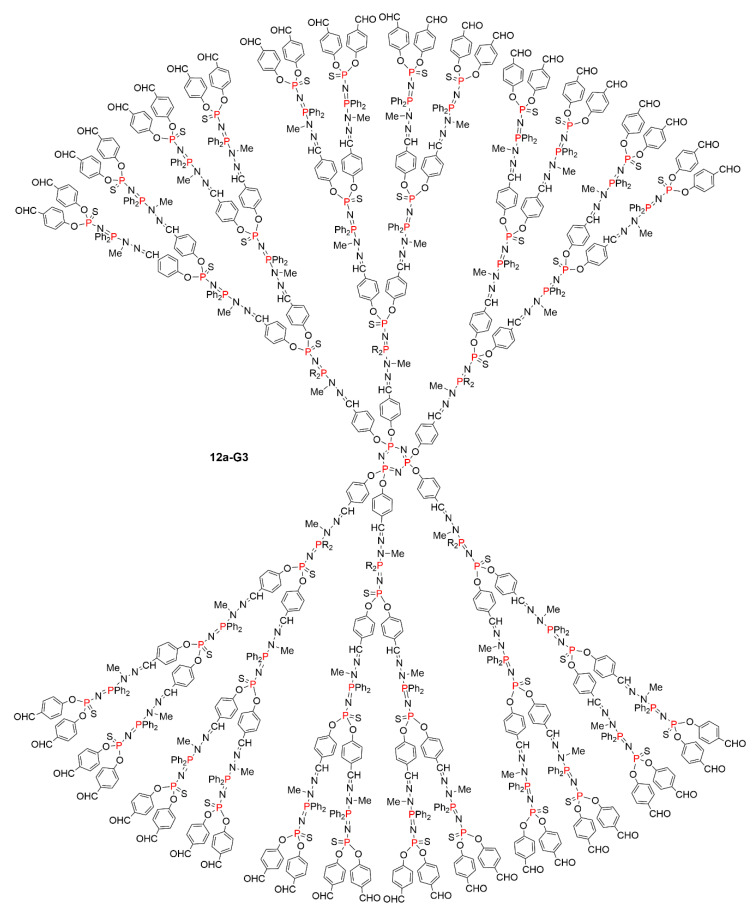
Full chemical structure of the dendrimer **12a-G3**, the linear form of which is shown in Scheme 12.

The first series of dendrimers incorporating the P=N-P=S linkage at each generation necessitated three steps to build a new generation. Starting from the aldehyde functions of an hexafunctional cyclotriphosphazene core, the first step was the condensation with methyl hydrazine, which afforded -N(Me)H terminal functions. The second step was the reaction with a chlorophosphine, which generated phosphine terminal functions. Two types of chlorophosphines were used, either diphenylchlorophosphine (**a**) or a chlorodiazaphospholane (**b**). The third step was the Staudinger reaction between these phosphines and the thiophosphine azide N_3_P(S)(OC_6_H_4_CHO)_2_ (**CD_2_**), which creates both the P=N-P=S linkages and a new level of branching points. This reaction afforded as terminal functions aldehydes on which the three-step reaction cycle could be repeated (Scheme 12). The synthesis was carried out up to the third generation **12a-G3** (Figure 7) [31].

**Scheme 13:**
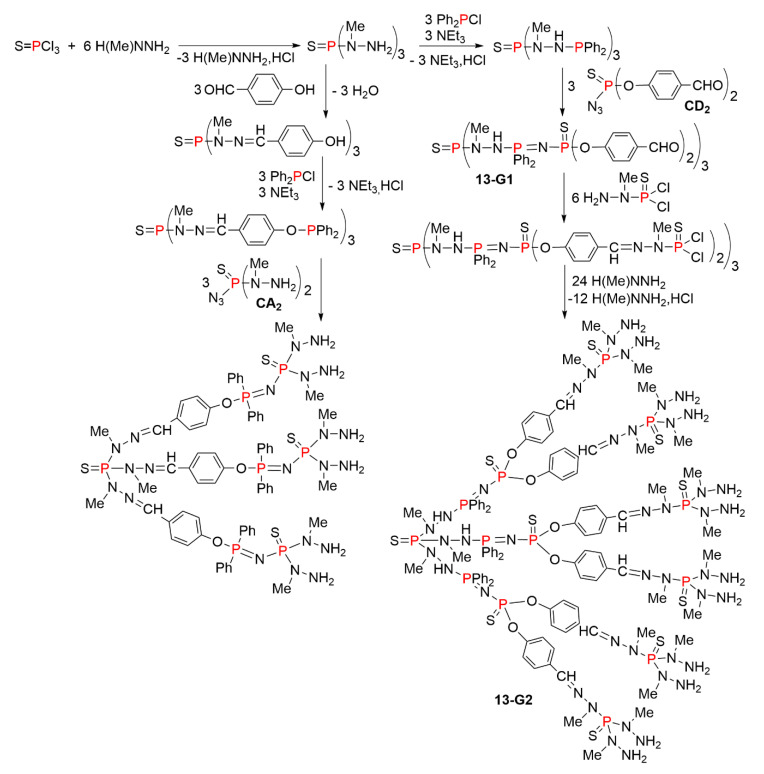
Synthesis of small dendrimers using the Staudinger process.

Smaller dendrimers were obtained using also the Staudinger process. Two methods are shown in Scheme 13, both starting with the synthesis of the thiophosphotrihydrazide S=P(NMeNH_2_)_3_. In a first attempt, the next step was a condensation reaction with hydroxybenzaldehyde, followed by the grafting of Ph_2_P to the phenol groups. The next and last step in this series was the Staudinger reaction with the thiophosphine azide N_3_P(S)(NMeNH_2_)_2_ (**CA_2_**). However, this reaction proceeded very slowly, even in refluxing THF, and it was impossible to totally avoid oxidation of the phosphine, thus the synthesis was stopped at this step. Another synthetic process was elaborated from the same thiophosphotrihydrazide, to which Ph_2_P was directly grafted. Only one Ph_2_P per NH_2_ group reacted. The reaction with the azido dialdehyde **CD_2_** proceeded easily at 0 °C, affording compound **13-G1**. Another process was used to continue the growing of the dendrimer, i.e., the condensation of the aldehydes with the phosphorhydrazide H_2_NNMeP(S)Cl_2_. The next step was the grafting of methylhydrazine on the P(S)Cl_2_ terminal functions. The purity of compound **13-G2**, having NH_2_ terminal functions, was not perfect, presumably because a small part of methylhydrazine (less than 10%) reacted with the P(S)Cl_2_ functions on the NH_2_ side instead of the desired NHMe side, thus the synthesis was stopped at this step [32].

**Scheme 14:**
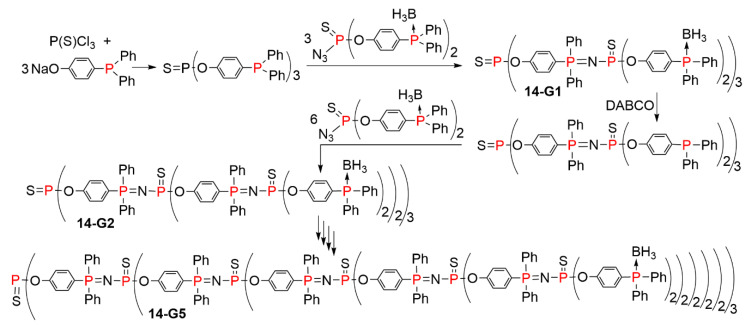
Synthesis of dendrimers using a single monomer at each step, for each generation.

**Figure 8:**
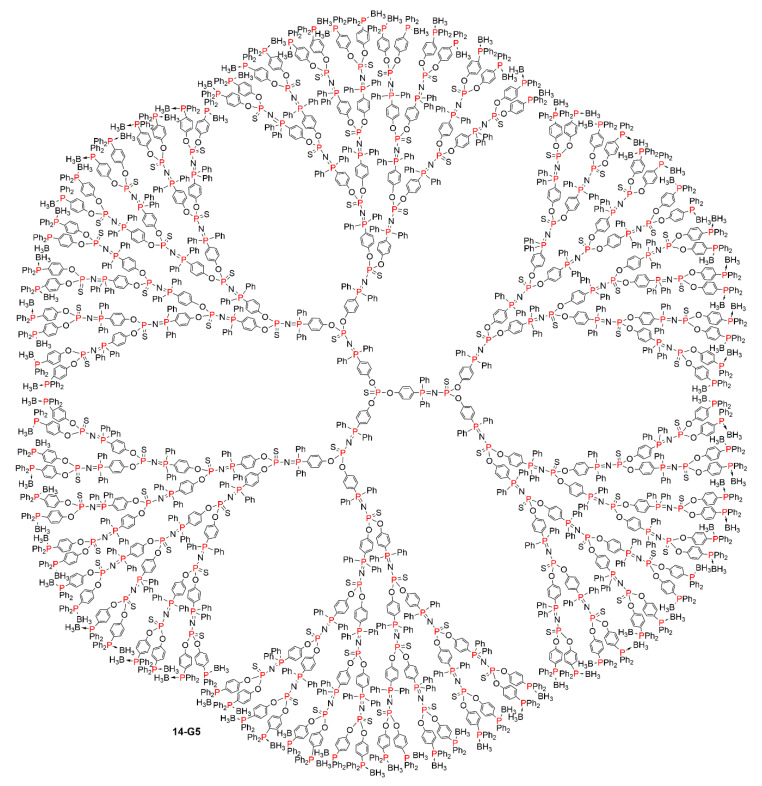
Full chemical structure of the dendrimer **14-G5** shown in a linear form in Scheme 14.

A two-step process using a single branching monomer for the Staudinger reaction was carried out up to the fifth generation. Starting from a triphosphine, the first step was the Staudinger reaction with an azidothiophosphine functionalized with two phosphines protected by BH_3_. The second step was simply the deprotection of the phosphines using DABCO (1,4-diazabicyclo(2.2.2)octane), to afford free phosphines, ready to react with the same azido monomer, as shown in Scheme 14. The Staudinger reaction and deprotection of the phosphines was carried out up to the synthesis of the fifth generation **14-G5**; its full structure is shown in Figure 8. Hyperbranched polymers having a related structure were obtained in a single step by deprotecting the monomer N_3_P(S)(OC_6_H_4_PPh_2_BH_3_)_2_ with DABCO. However, comparison of the physical properties of these hyperbranched polymers and of the corresponding dendrimers, both having analogous molecular weights, pointed out large differences [33].

**Figure 9:**
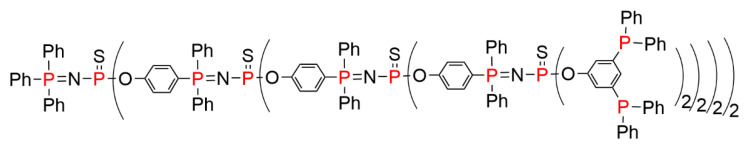
Another dendrimer synthesized as shown in Scheme 14, starting from triphenyl phosphine as the core.

Exactly the same process was applied later to triphenylphosphine used as core. The synthesis was carried out up the third generation, functionalized by diphosphines on the surface, as shown in Figure 9 [34].

All the processes shown in Scheme 12 and Scheme 13 are relatively lengthy. After these first attempts, the Staudinger reaction was used to afford accelerated methods of synthesis of dendrimers, in which one step affords one generation. A review has gathered several types of accelerated methods for the synthesis of phosphorus dendrimers, but it was not focused on Staudinger reactions [35].

**Scheme 15:**
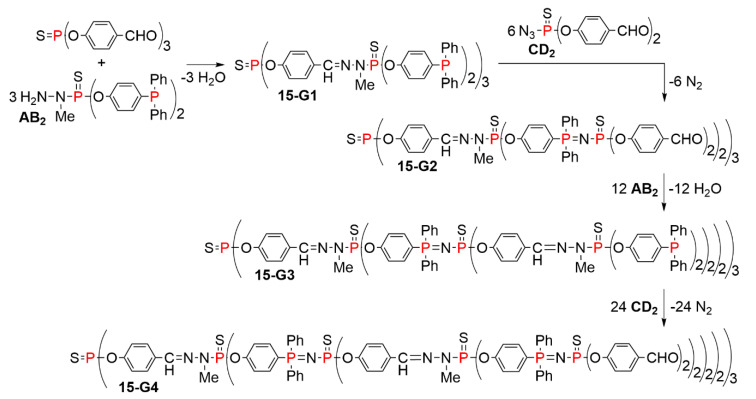
Synthesis of layered dendrimers using two branched monomers of types **AB_2_** and **CD_2_**. Only one step is needed for the synthesis of one generation.

**Figure 10:**
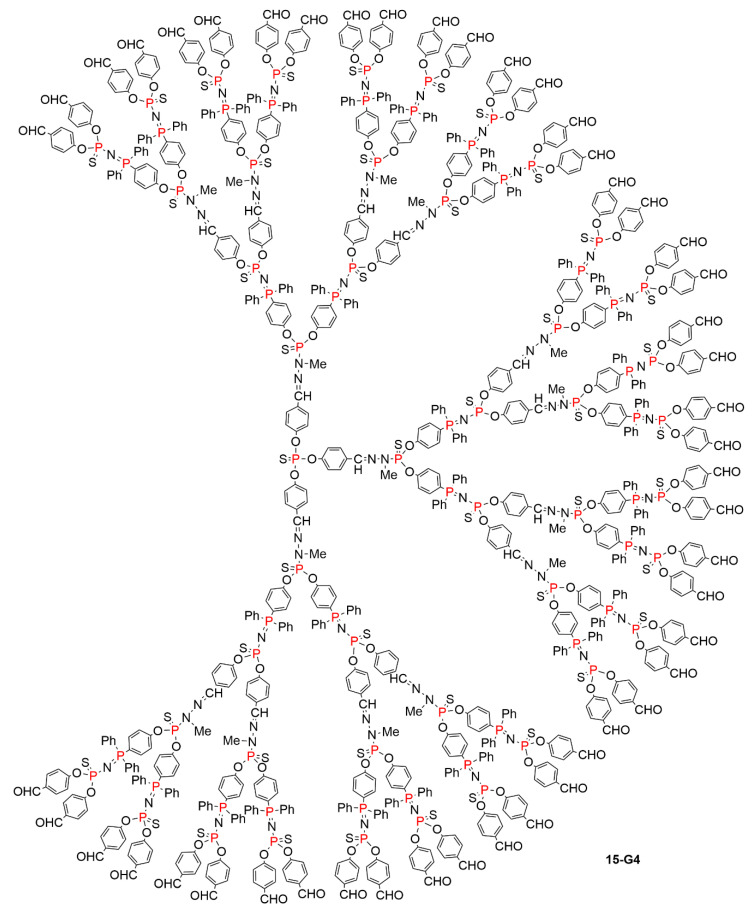
Full chemical structure of the layered dendrimer **15-G4**, for which the linear structure is shown in Scheme 15.

A first attempt was carried out using two branched monomers named **AB_2_** and **CD_2_**, in which **A** is an amine, suitable to react with **D**, which is an aldehyde, and **B** is a phosphine suitable to react with **C**, which is an azide. The fourth generation of this family of dendrimers, **15-G4**, was obtained in only four quantitative steps. No purification was needed, as the by-products are only N_2_ and H_2_O (Scheme 15). The synthesis of the fourth generation **15-G4** was also carried out one-pot, by adding sequentially strictly the stoichiometric amounts of reagents needed at each step. The purity of the fourth generation obtained by isolation after each step or synthesized one-pot was found to be very similar. The full structure of dendrimer **15-G4** is shown in Figure 10. It can be seen that this family of compounds are layered dendrimers, which means that the same type of terminal functions is obtained with each two generations, contrarily to each generation for classical dendrimers. The terminal functions are alternatively either phosphines or aldehydes [36].

**Scheme 16:**
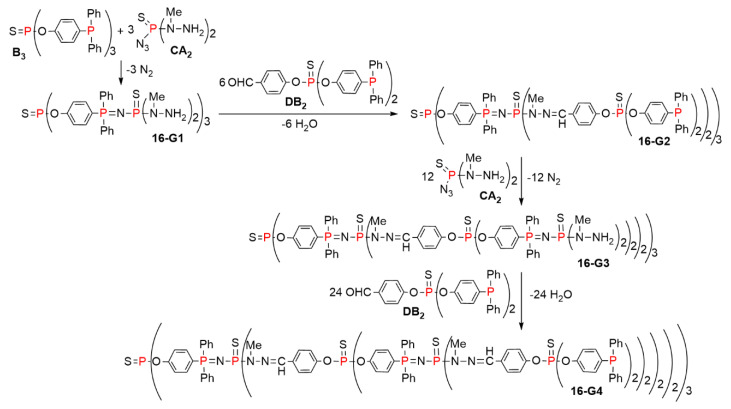
Synthesis of layered dendrimers based on the reaction with the **CA_2_** and **DB_2_** branched monomers.

This concept was then expanded to the use of other types of complementary branched monomers. For this purpose, the branched monomers **CA_2_** and **DB_2_** were synthesized, where **A**, **B**, **C**, and **D** are the same functional groups than in Scheme 15. These monomers were used for the synthesis of another type of layered dendrimer, up to the fourth generation **16-G4**, as shown in Scheme 16. The terminal functions were alternatively either hydrazine or phosphines, and the by-products were either N_2_ or H_2_O, as in the previous case [37].

**Scheme 17:**
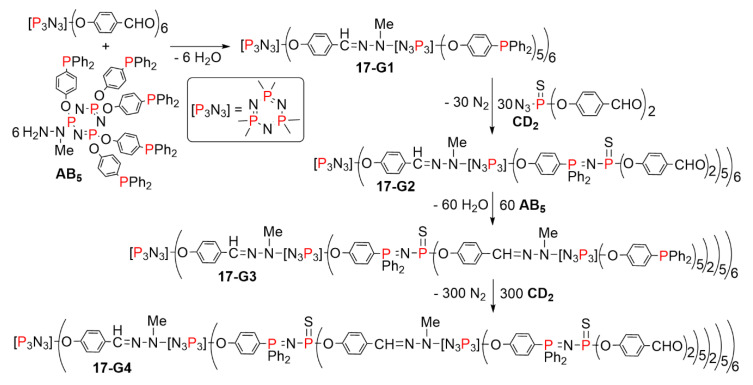
Synthesis of layered dendrimers based on the reactions with the **AB_5_** and **CD_2_** branched monomers.

Using the same principle, highly branched monomers of types **AB_5_** and **CD_5_** were synthesized thanks to the specific reactivity of the cyclotriphosphazene [N_3_P_3_], in which it is possible to differentiate the reactivity of one function from that of the five other functions [38]. In a first attempt, the crowded **AB_5_** monomer based on [N_3_P_3_] was used together with the **CD_2_** monomer, as shown in Scheme 17. The synthesis was carried out up to the fourth generation. The use of the **AB_5_** monomer at two steps induced a very large increase of the number of terminal functions. Indeed, the fourth generation **17-G4** possesses 600 aldehyde terminal functions, to be compared with only 48 aldehyde terminal functions for the fourth generation **15-G4** [39].

**Scheme 18:**
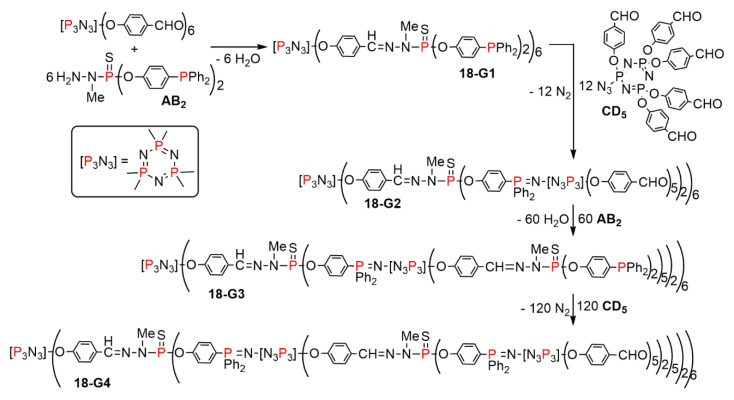
Synthesis of layered dendrimers based on the reactions with the **AB_2_** and **CD_5_** branched monomers.

**Figure 11:**
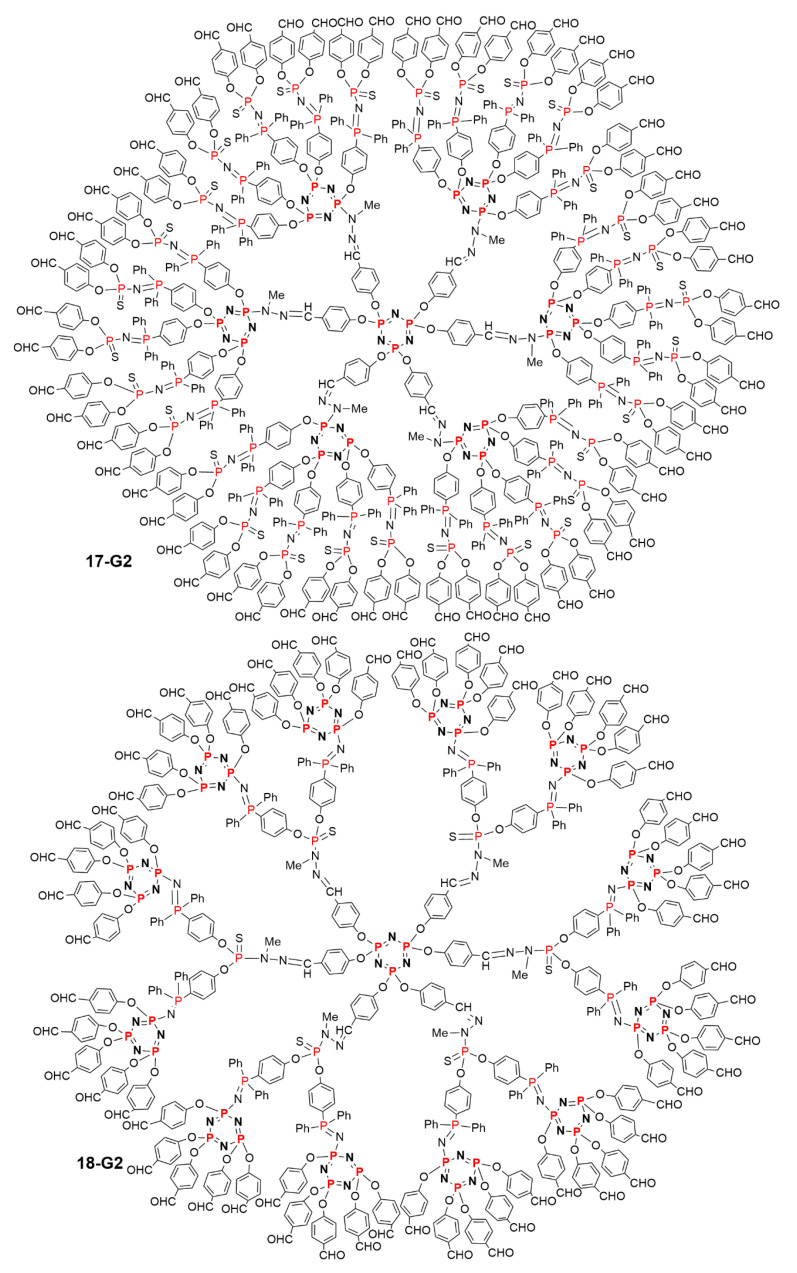
Comparison between the full structure of the second-generation dendrimers **17-G2** and **18-G2**.

The reaction of the crowded **CD_5_** monomer based on [N_3_P_3_] used together with the **AB_2_** monomer was also studied, as shown in Scheme 18. With this large monomer **CD_5_**, the phosphazene linkage induced by the Staudinger reaction was stabilized by N_3_P_3_ instead of P=S when using the **CD_2_** monomer. The synthesis was also carried out as previously shown up to the fourth generation of the layered dendrimer **18-G4**. As in the previous case, the use of the branched monomer **CD_5_** at two steps induced a very large increase of the number of terminal functions. Dendrimer **18-G4** was obtained in four steps. It also bears 600 aldehyde terminal functions, as the dendrimer **17-G4** [39]. Despite their similarity, the families of dendrimers **17** and **18** are intrinsically different, as the crowding due to the highly branched monomers is located in different areas, as illustrated by the comparison between the full structures of dendrimers **17-G2** and **18-G2**, both possessing 60 aldehyde terminal functions (Figure 11).

**Scheme 19:**
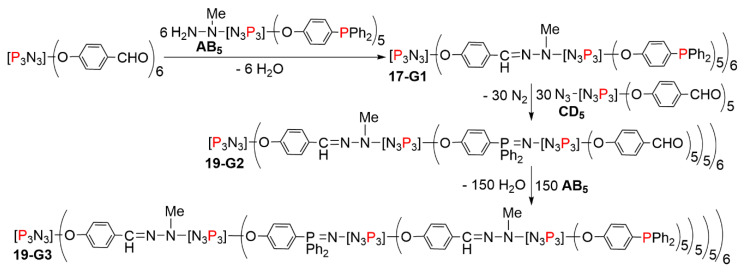
Synthesis of the third generation dendrimer **19-G3** in only 3 steps, functionalized with 750 phosphine terminal functions.

**Figure 12:**
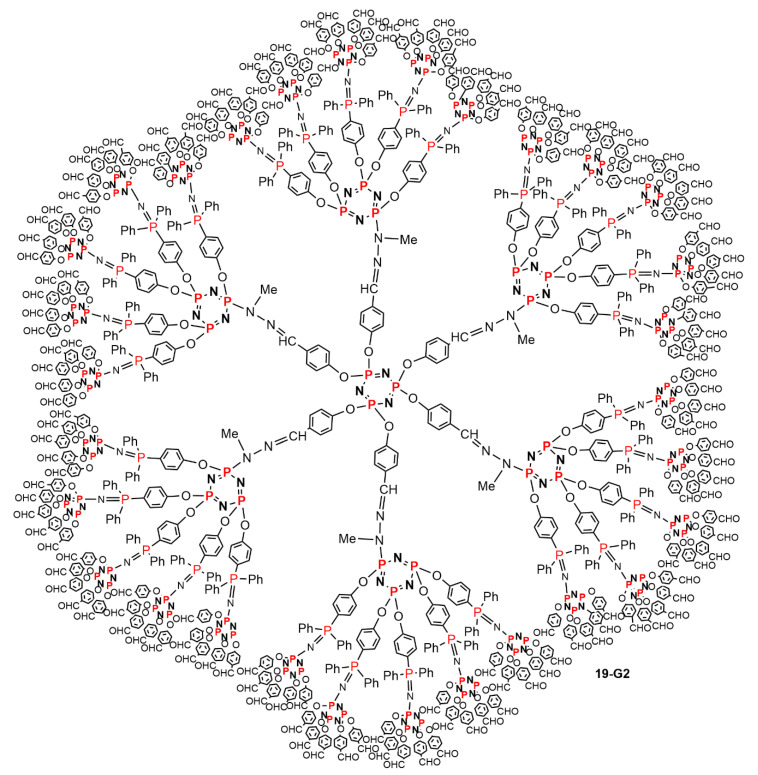
Full chemical structure of dendrimer **19-G2**, functionalized with 150 aldehyde terminal functions, built with **AB_5_** and **CD_5_** monomers, for which the linear structure is shown in Scheme 19.

In a final attempt, a dendrimer was synthesized using both crowded monomers **AB_5_** and **CD_5_**, both based on the cyclotriphosphazene. The first step was identical to the first step shown in Scheme 17, affording compound **17-G1**, functionalized with 30 phosphine terminal functions. The second step was the reaction with the **CD_5_** monomer, affording the second-generation dendrimer **19-G2** functionalized with 150 aldehyde terminal functions. The third step was the reaction with the **AB_5_** monomer, affording the third-generation dendrimer **19-G3** functionalized with 750 phosphine terminal functions (Scheme 19). Due to the very large crowding of this dendrimer, the reaction with the **CD_5_** monomer to get the next generation was not attempted. Figure 12 displays the full structure of the second generation **19-G2**. It is not possible to draw the full structure of the third generation, due to very large overlap between the terminal functions in a 2-dimentional representation. There is no other report to date for obtaining so large a number of terminal functions in a dendrimer, using only 3 steps for its synthesis [39].

### 5.2. The Usefulness of P=N-P=S Linkages for the Synthesis of Sophisticated Dendritic Architectures

The P=N-P=S linkages generated by the Staudinger reaction are particularly useful for the synthesis of dendrimers, as shown in the previous paragraphs, as they afford stable compounds. However, the charge delocalization along this linkage induces the presence of a negative charge on the S atom, larger than in the case of other P=S groups, not included in such linkages [40]. This particularity allows us to anticipate a versatile reactivity on the P=S groups of P=N-P=S linkages, such as alkylation or complexation. Both have been studied, not with regular dendrimers, but with dendrimers incorporating the P=N-P=S linkages at a single layer or at a few layers inside the structure.

**Scheme 20:**
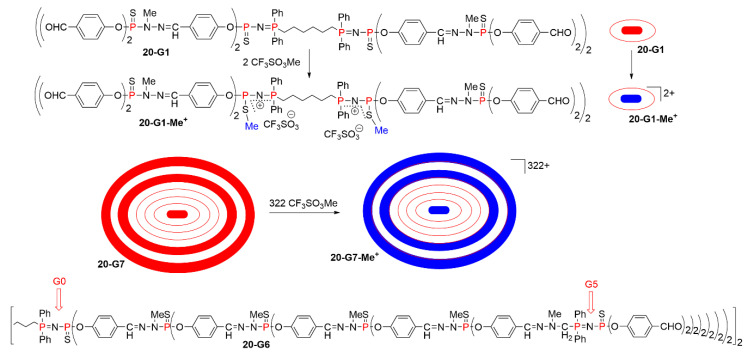
Alkylation on sulfur of P=N-P=S linkages of a first-generation dendrimer, and schematization of the same reaction on a generation 7 dendrimer, having 2 P=N-P=S linkages at the core, 64 at the level of the fifth generation, and 256 at the seventh generation. The structure of **20-G6** showing the place of two layers of P=N-P=S linkages.

The alkylation was carried out first with methyltriflate. Dendrimers built from a diphosphine at the core and having two P=N-P=S linkages linked to this core, 64 at the level of the fifth-generation, and 256 at the level of the seventh-generation were synthesized up to this generation, terminated with 512 aldehyde groups. All these dendrimers from **20-G1** to **20-G7** were reacted with a stoichiometric amount of CF_3_SO_3_Me, as shown in Scheme 20. The methylation induced a dramatic shift of the signal of the P=N-P=S linkages in the ^31^P NMR spectra. For instance, for the first generation with aldehyde terminal functions, the system of two doublets at 20.6 ppm (Ph_2_P=N-) and 53.2 ppm (-P=S, ^2^J_pp_ = 36 Hz) was replaced by another system of two doublets at 28.8 ppm (-P-*S*-Me) and 32.9 ppm (Ph_2_P=N-, ^2^J_pp_ = 18 Hz). The singlet corresponding to P=S groups not included in P=N-P=S linkages is not modified (δ = 62.3 ppm before and after addition of methyltriflate). The same behavior was observed with all the other dendrimers upon methylation [41]. The same family of dendrimers was also reacted with other triflates such as allyl and propargyl triflates; both induced alkylation only on the sulfur of P=N-P=S groups [42].

**Scheme 21:**
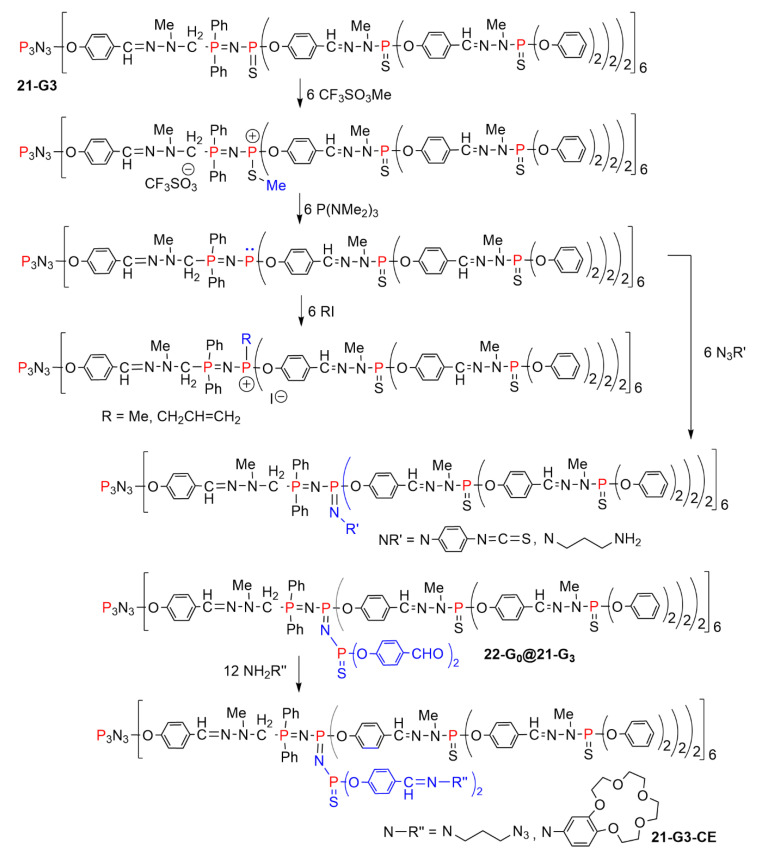
Different types of reactions carried out on the P=N-P=S linkages in the internal structure of dendrimer **21-G3**.

**Figure 13:**
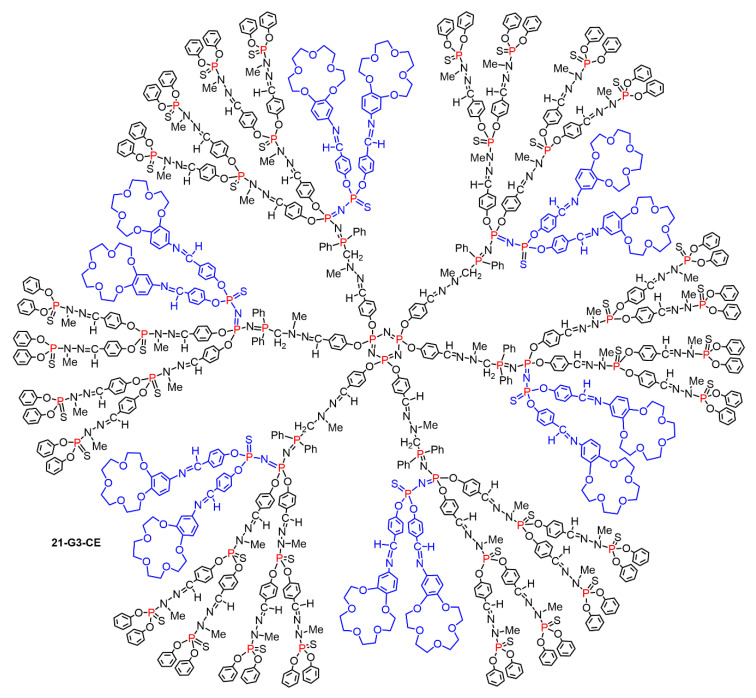
Full structure of the dendrimer **21-G3-CE** bearing 12 crown ether derivatives inside its structure.

Alkylation of the P=S groups induces a weakening of the bond between phosphorus and sulfur, which can be cleaved with a nucleophilic phosphine such as P(NMe_2_)_3_. Such a reaction generates highly reactive trivalent phosphorus atoms (P^III^) inside the structure of dendrimers. A versatile reactivity was developed from these P^III^ atoms. Reaction with methyl or allyl iodide generated phosphonium salts at precise layers inside the structure. However, the most important reaction was the Staudinger reaction with functionalized azides, which permitted the grafting of isothiocyanate, primary amine, or aldehyde internal functions, while generating P=N-P=N-P=S linkages. These reactions were carried out with several dendrimers, but are illustrated in Scheme 21 only on the third-generation dendrimer **21-G3**, having 6 P=N-P=S linkages in its structure. The aldehydes of dendrimer **22-G0@21-G3** were then used in condensation reactions with amines, leading to the internal grafting of azides or crown ethers (**21-G3-CE**, Figure 13) [43].

**Scheme 22:**
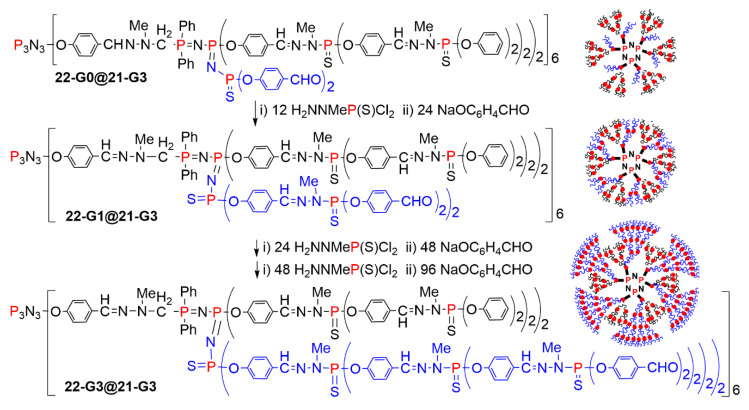
Synthesis of branches inside a dendrimer, up to **22-G3@21-G3**.

**Figure 14:**
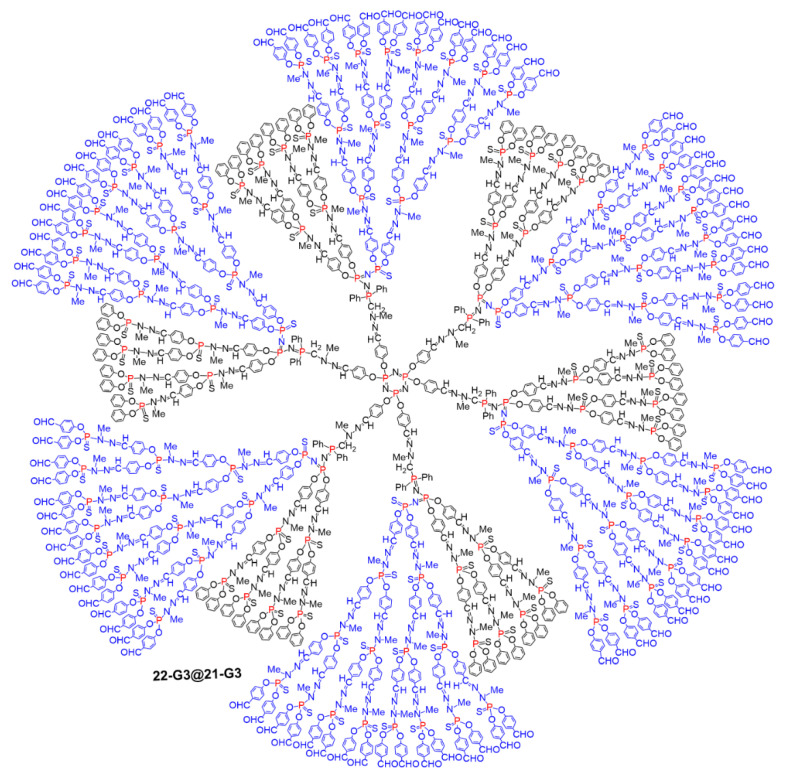
Full structure of dendrimer **22-G3@21-G3**. The newly synthesized branches are represented in blue.

The presence of aldehyde functions inside the structure of dendrimers enabled the growing of new branches from inside, leading to a very sophisticated type of dendritic structure. Two different methods have been used to generate these new branches. One method used the classical way for the synthesis of phosphorus dendrimers [44,45,46] up to the twelfth generation [47], i.e., the condensation of the phosphorhydrazide H_2_NNMeP(S)Cl_2_ with the aldehydes, and the substitution of chlorine atoms with hydroxybenzaldehyde in basic conditions. This method was applied for the growing of internal branches up to the third generation, as shown in Scheme 22 and Figure 14 [48].

**Scheme 23:**
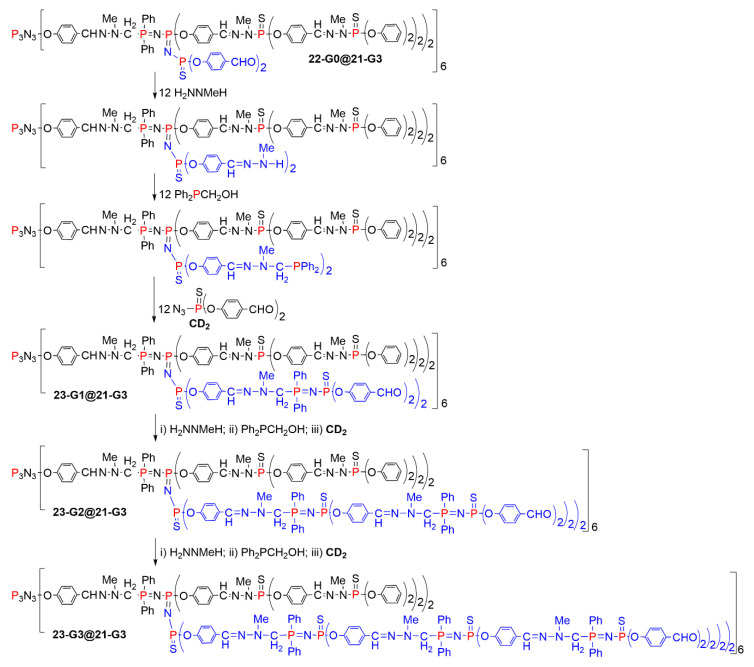
A three-step method for growing new branches inside a dendrimer, up to **23-G3@21-G3**.

The second method for growing new branches starting from the dendrimer **22-G0@21-G3** necessitated three steps to build one generation. The first step was the condensation of methylhydrazine with the internal aldehydes. The second step was a reaction between the NH internal functions and the phosphine Ph_2_PCH_2_OH used in excess and upon heating. The third step was the Staudinger reaction with the **CD_2_** azido dialdehyde, to afford the first generation of internal branches, namely compound **23-G1@21-G3**. This three-step process was again carried out twice, to afford finally the dendritic structure **23-G3@21-G3** (Scheme 23) [48]. It should be emphasized that with both methods shown in Scheme 22 and Scheme 23, the new branches are longer than the branches of the initial dendrimer (**21-G3**), and they are in both cases functionalized by aldehyde terminal functions.

**Scheme 24:**
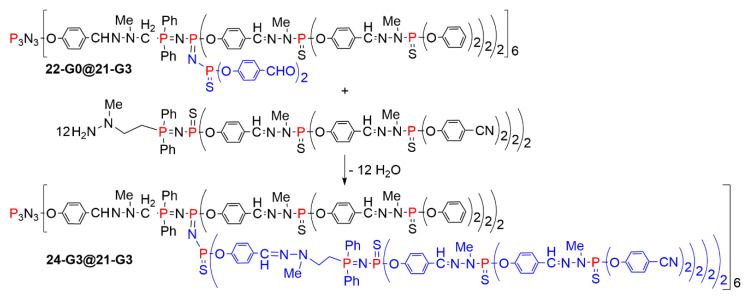
Grafting of 12 dendrons inside the structure of a dendrimer, up to **24-G3@21-G3**.

Later on, attempts were made to directly graft a dendron having an amine at the core to be reacted with the internal aldehydes. It was possible to graft a second generation dendron to create the third generation, but the reaction needed 10 days to go to completion (Scheme 24) [49]. This reaction afforded in a single step the dendritic structure **24-G3@21-G3**, analogous to that of dendrimer **22-G3@21-G3**.

**Scheme 25:**
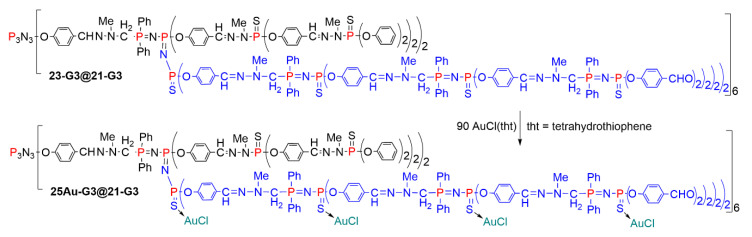
Selective complexation of gold by P=N-P=S and P=N-P=N-P=S linkages.

Besides the alkylation of the sulfur atom, the P=N-P=S linkages are suitable for the complexation of metals, in particular of gold [50]. Several of the dendritic structures shown in the previous paragraphs incorporating P=N-P=S linkages in some parts of their structure were used for the complexation of gold. Only one example is shown in Scheme 25. It concerns the dendritic structure **23-G3@21-G3**, in which only the new branches, containing P=N-P=S (or P=N-P=N-P=S), are able to complex gold. The complexation after reaction with Au-Cl(tht) (tht = tetrahydrothiophene) was easily detected by ^31^P NMR. Indeed, before complexation the P=N-P=S linkages of compound **23-G3@21-G3** are characterized by two doublets at ca. 13 ppm (P=N) and ca. 52 ppm (P=S), with ^2^J_PP_ = 31 Hz. These doublets disappeared after complexation, on behalf of the appearance of two other doublets at ca. 14 ppm (P=N) and ca. 35 ppm (P=S-Au), with ^2^J_PP_ = ca. 18 Hz for compound **25Au-G3@21-G3**. The signal corresponding to the P=S groups not included in P=N-P=S linkages did not change (ca. 62 ppm before and after addition of AuCl(tht)), indicating that they were not involved in the complexation [51].

**Scheme 26:**
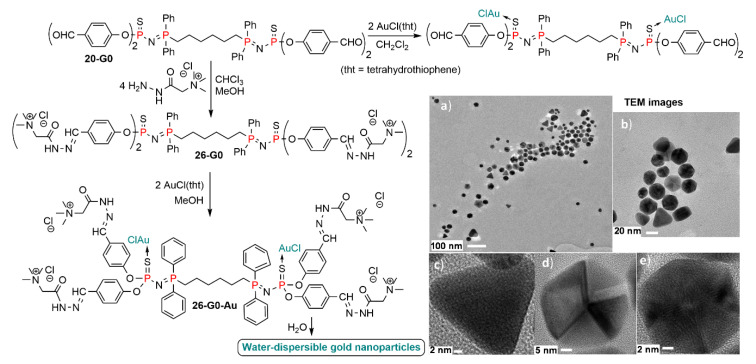
Complexation of gold by the small **26-G0** dendrimer and TEM images of gold nanoparticles generated only by addition of water to the dendrimer complex (images from Ref. [52], copyright Caminade et al., open access article).

The same type of complexation was recently carried out with a small dendrimer of the **20-Gn** family. It was reacted with the Girard’s T reagent to afford the water soluble derivative **26-G0**. Reaction with AuCl(tht) afforded as expected the corresponding complex **26-G0-Au** (Scheme 26). This compound is a white solid, stable for at least several months in the solid state, which afforded colorless solutions in organic solvents. However, a red solution was obtained when dissolving this dendrimer complex in water. Analysis of this water solution by TEM (transmission electron microscopy) revealed the presence of gold nanoparticles. High resolution TEM (images c, d, and e in Scheme 26) showed that the form of all nanoparticles was either triangles or associated triangles of gold atoms. The dendrimer **26-G0-Au** acted simultaneously as a mild reducer (Au^I^ −> Au^0^) and as a nanoreactor, favoring the self-assembly of gold atoms and promoting the growth and stabilization of isolated gold nanoparticles. The reduction is probably due to the release by hydrolysis of the hydrazine bond of a small quantity of the Girard’s T reagent, able to act as a reducing agent. This is an unprecedented method for the synthesis of colloidal suspensions of water-soluble gold nanoparticles [52].

## 6. Conclusions

We have shown in this review that trivalent phosphorus derivatives are useful reagents for the synthesis of dendrimers and of very original dendritic structures. The main limitation to their use is their easy oxidation by oxygen when exposed to air. Indeed, it is impossible to purify a dendrimer in which one or a few functions would have been oxidized, thus even traces of oxygen have to be avoided. To limit this problem, reactions are generally carried out under inert gases, and triarylphosphines are preferred over trialkylphosphines, which are more easily oxidized. Among the three main methods of dendrimer synthesis using trivalent phosphorus derivatives that have been proposed, i.e., the alkylation of phosphines, the use of phosphoramidate reagents, and the Staudinger reaction of phosphines with azides, the last two methods have been the most widely used, and possess certainly the largest potential for the future. Besides the synthetic aspects, a few practical uses have been proposed with these particular dendrimers. One can cite in particular catalysis with phosphine complexes [8,9], materials with the elaboration of gold nanoparticles via an unprecedented method [52], and diverse aspects of biology, such as potential fluorescent labels [27], PCR primers having a profound effect on the PCR products mobility [17], or efficient inhibitors of cancer cell growth [19]. Undoubtedly, the study of properties of dendrimers based on the reactivity of trivalent phosphorus derivatives constitutes the main challenge for the future of these special dendrimers.

## Data Availability

No new data were created or analyzed in this study. Data sharing is not applicable to this article.

## Sample Availability

No compounds are available from the authors.
